# Leveraging the Academic Artificial Intelligence Silecosystem to Advance the Community Oncology Enterprise

**DOI:** 10.3390/jcm12144830

**Published:** 2023-07-21

**Authors:** Kevin J. McDonnell

**Affiliations:** Center for Precision Medicine, Department of Medical Oncology & Therapeutics Research, City of Hope Comprehensive Cancer Center, Duarte, CA 91010, USA; kemcdonnell@coh.org

**Keywords:** artificial intelligence, City of Hope, oncology, community practice

## Abstract

Over the last 75 years, artificial intelligence has evolved from a theoretical concept and novel paradigm describing the role that computers might play in our society to a tool with which we daily engage. In this review, we describe AI in terms of its constituent elements, the synthesis of which we refer to as the AI Silecosystem. Herein, we provide an historical perspective of the evolution of the AI Silecosystem, conceptualized and summarized as a Kuhnian paradigm. This manuscript focuses on the role that the AI Silecosystem plays in oncology and its emerging importance in the care of the community oncology patient. We observe that this important role arises out of a unique alliance between the academic oncology enterprise and community oncology practices. We provide evidence of this alliance by illustrating the practical establishment of the AI Silecosystem at the City of Hope Comprehensive Cancer Center and its team utilization by community oncology providers.

## 1. Introduction

Artificial intelligence (AI) plays an ever-increasing role in our daily lives most immediate to us in our use of entertainment, consumer and communication products [1,2]. Less immediately obvious to the oncology patient, AI has become an important tool to assist the clinical management of and guide therapy for cancer [3,4,5]. Within the academic oncology sphere, AI already has a significant impact. For example, AI has substantial, established roles in precision oncology [6,7,8], clinical oncology decision-making [9,10,11], digital cancer pathology [12,13,14,15,16] and radiology [17,18,19]. For community oncology practice, the role of AI remains limited but continues to emerge [20,21,22]. In this review, we seek to further expand knowledge of the role that AI plays in the community practice of oncology. We organize this manuscript into two parts. In Part I, we review the history, current state and emerging innovations relating to the computer hardware, data and software components that make AI possible. For conceptual simplicity and coherence, we refer to the synthesis of these components as the AI Silecosystem. We trace the emergence of the AI Silecosystem, its current state and future directions within the context of a Kuhnian scientific paradigm. In Part II, we provide a case example of the establishment and application of the AI Silecosystem in community oncology practice. We review the historical role and current integral position that academic medical institutions occupy in facilitating utilization of the AI Silecosystem by the community oncologist. We describe and place special emphasis on our experience at the City of Hope COH) Comprehensive Cancer Center to advance community oncology team utilization of the AI Silecosystem.

## 2. The AI Silecosystem as Kuhnian Paradigm

**Figure 1:**
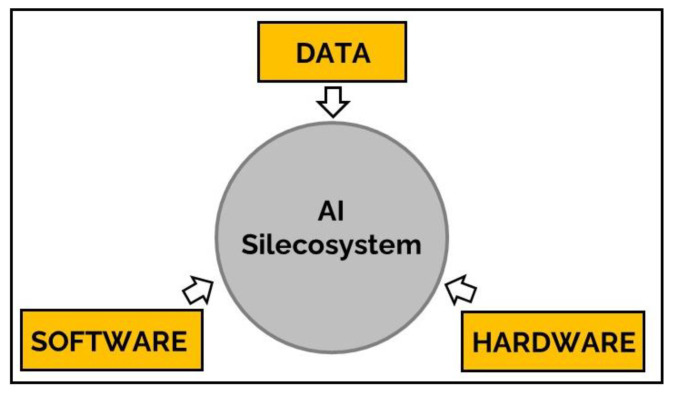
**The AI Silecosystem comprises hardware, data and software components.** The integrated components of the AI Silecosystem facilitated the development, utilization and evolution AI.

By AI Silecosystem we mean the synthesis of data, hardware and software that undergird the operation, make available the use, and fuel the growth of AI (Figure 1). To conceptually appreciate the history, progress and future trajectory of the AI Silecosystem, we may conceive and provide description of the AI Silecosystem as a Kuhnian paradigm [23]. As a Kuhnian paradigm, the AI Silecosystem has disrupted and shifted the original paradigm of computer as finite *computational* machine to the novel paradigm of computer as versatile, multipotent *thinking* machine. This paradigm shift characteristically matures through three discrete, iterative stages: inception, intermission and invigoration.

## 3. Origins of the AI Silecosystem: A Chronicle of an Emergent Paradigm

### 3.1. Inception: Articulation Anticipates Actualization

**Figure 2:**
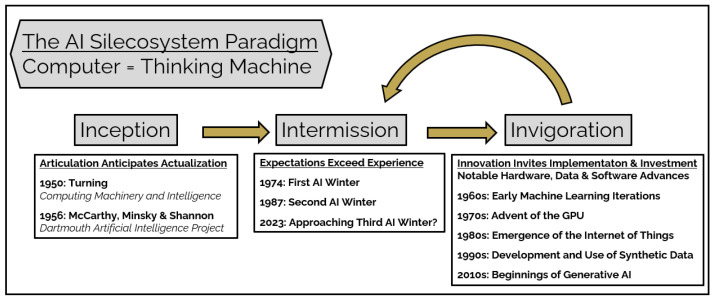
**The AI Silecosystem paradigm conceives of the computer as thinking machine.** As proposed by Turning [27] and McCarthy et al. [28], the computer may function as bone fide thinking machine, rather than mere computational machine. In accordance with a Kuhnian paradigm, the AI Silecosystem undergoes a series of stages: Inception, Intermission and Invigoration. Characteristically, the paradigm experiences a series of iterative Intermission and Invigoration cycles as new expectations develop and innovations occur.

McCulloch and Pitts defined the incipient notion of computer as a thinking machine, suggesting that engineers might design computers to functionally mimic the operation of the human nervous system. In this theoretic nervous system model, an individual neuronal logic element achieves its ultimate activation state through cumulative summation of weighted inputs generated from a syndicate of contiguous neuronal logic elements [24]. This proposal represented an important architectural anlage preceding physical construction of Rosenblatt’s early neural network, the Perceptron [25,26]. Rosenblatt’s Mark 1 Perceptron neural network machine demonstrated the ability to perform basic visual pattern recognition. These early insights and accomplishments gave rise to an inchoate AI Silecosystem that Alan Turing further accelerated with his proposition that machines might “think” through serial adjudication of true and false logic states [27] (Figure 2). Formal AI development acquired significant academic interest and gained further momentum in 1956 when the early pioneers, McCarthy, Minsky and Shannon, convened a summer research convention at Dartmouth College where they sought critical evaluation of the assertion that “every aspect of learning or any other feature of intelligence can in principle be so precisely described that a machine can be made to simulate it” [28]. Historians credit McCarthy as one of the originators of the term “artificial intelligence”. Consistent with previous Kuhnian paradigms, articulation of the AI Silecosystem paradigm anticipated its practical implementation.

### 3.2. Intermission: Expectations Exceed Experience

Initial efforts to create and implement the AI Silecosystem experienced setbacks. Between 1970 and 1990, a series of pivotal, adverse events led to intermittent intermissions in AI Silecosystem utilization, research and development. The inability of the AI Silecosystem to deliver its promise to perform complex, traditionally human-only tasks such as language translation, speech recognition and advanced image analysis efficiently and accurately muted expectations for AI-based approaches. These shortcomings prompted sponsors to withdraw financial support from several prominent AI initiatives. During this two-decade period, the Defense Advanced Research Projects Agency (DARPA) reduced funding for Carnegie Mellon’s AI speech recognition program, and the United States National Research Council ended its financing of AI language translation efforts [29]. Following the Lighthill report, the United Kingdom halted further public AI development [30], and Japan curtailed AI investment after its Fifth Generation project failed to meet its articulated goals [31]. These setbacks instigated widespread public disillusionment with AI and precipitated a series of intermissions in further AI discovery and advancement, that is, the “AI Winters”. Intermissions, such as the AI Winters, Kuhn would recognize as expected phases in the lifecycle of a paradigm shift. Full acceptance of a paradigm often must await creation of the technology and evaluation tools to permit complete use, valid assessment and thorough validation of the novel paradigm. Kuhn notes, for example, that many years passed after Newton and Einstein first introduced their mechanics and relativity paradigms until the availability of experimental verification protocols allowed scientists to fully understand, confirm and accept their revolutionary ideas [23]. Ultimately, innovation and insight facilitate endorsement and adoption of emerging paradigms, and, specifically, in the case of the AI Silecosystem, led to thawing of the AI Winters.

### 3.3. Invigoration: Innovation Invites Implementation and Investment

Innovation of and transformational progress within three core elements of the AI Silecosystem, i.e., computer hardware, data acquisition and processing and software algorithms, hastened thawing of the AI Winters. The following sections survey these key, instrumental innovations and advances.

#### 3.3.1. Advances in Computer Hardware: The Engines That Power the AI Silecosystem

If we view the AI Silecosystem as a computational vehicle, its hardware elements function as the engines powering AI algorithmic processing. The invention of the silicon chip [32], introduction of multicore constructs [33] and development of ultrahigh capacity data storage systems [34], among other hardware innovations, enabled efficient, inexpensive performance of computationally complex, data-dense AI algorithms. The following more recent advances promise to further boost adoption and expansion of the AI Silecosystem.

##### Quantum Computing

Quantum computing uses the quantum bit (qubit) as its fundamental unit of information in contrast to conventional digital computing which employs the binary bit. Two different value states define the classic binary bit, and these value states exhibit mutual exclusivity (either 1 or 0). The qubit, however, may retain both values states simultaneously (1 and 0) in a quantum condition known as superposition. Superposition enables more rapid completion of complex, intensive computational tasks by quantum computation; digital computation cannot complete these tasks within a meaningful time frame. The computational superiority of the quantum computer, termed “quantum supremacy”, was first demonstrated by Google in 2019 using a programable superconducting processor [35]. Quantum supremacy has the potential to amplify the power and practical utility of the AI Silecosystem. For example, computational scientists have developed and now apply AI algorithms to solve complicated combinatoric problems such as those encountered in molecular oncology drug design [36] and cancer diagnostics [37]. Processing of such AI algorithms on traditional computer platforms, however, might require exorbitant, cost- and time-prohibitive computational resources; implementation of quantum computation may allow tractable, economic solutions for combinatoric and other equally complex oncologic questions. Oncologists have successfully used quantum computing, together with AI applications, in the prediction of breast cancer [38], the application of radiotherapy [39] and cancer histologic assessment [40].

##### Artificial-Intelligence-Boosted Internet of Things (AIoT)

The internet of things (IoT) describes a system of local and remote physical instruments with communication, data processing, computational, memory storage and sensor capabilities interconnected via the internet and/or a local network [41,42]. The IoT aims to leverage the full potential of modern digital resources to optimize and assist with the activities and pursuits of daily living. Domestic examples of the IoT include smart speakers, home security systems and integrated, residential thermostat devices. The IoT has the potential for broad societal utilization. Specifically, within the sphere of health care, the IoT, i.e., the internet of medical things (IoMT), has enabled new, vital medical services, for instance, distance clinical assessment and monitoring [43,44] and remote health emergency notification [45]. In addition, investigators have proposed using the IoMT to enhance breast cancer detection [46], patient-centric healthcare [47,48], and the performance of health-care-related deep learning models [49].

With the advent of AI, the next iteration of the IoT emerged: artificial-intelligence-boosted IoT (AIoT) [50]. The AIoT underpins a range of familiar IoT applications such as autonomous driving vehicles [51], industrial robots [52] and surveillance drones [53]. The AIoT has provided impetus for several AI-based initiatives, for example, the development of anticipatory manufacturing machine maintenance, automated optimization of commercial operational efficiency and machine-learning-based urban safety monitoring and traffic control. Hospitals have begun using the AIoT to maintain efficient daily facility functioning and provide centralized patient monitoring. At COH, researchers have harnessed the AIoT to ensure safe, timely and effective post-surgery recovery for the patient after return to their home [54].

##### Distributive Edge Computing

Shared, centralized high-performance computer centers (HPCCs) have made available to a multitude of scientists the computer resources required to perform highly complex, computationally intense analyses. A HPCC may be located at a significant physical distance from the data source; moreover, as a shared resource, HPCC analytic jobs enter a work queue and process them in a serial fashion. The geographical and operational architecture of the HPCC results in “in due time” job completion. A complementary data analytic approach, edge computing, redistributes data processing, computations and memory storage from HPCC hubs to smaller, local computer nodes contiguous with the data source [55]. Edge computer nodes excel at “now time” processing of smaller discrete data parcels. For certain applications, most notably IoT platforms, edge computing offers distinct advantages over centralized HPCC processing: improved efficiency, low latency and increased agility; further, for large institutions, with often immensely large HPCC computational demands, edge computing helps alleviate computational backlog and obviate compromise of network bandwidth. Currently, edge computing plays an indispensable role in healthcare, processing data originating from local clinics as well as patient wearable monitoring devices. [56,57]. Researchers have begun to leverage the AI Silecosystem to catalyze new discoveries in and applications of edge computing. Recent efforts seek to bring the power, versatility and efficacy of AI to the edge in order to enhance local analytic capabilities [58,59,60]; specific initiatives seek to apply AI to edge immune-oncology and precision oncology computational efforts [61,62].

##### Cloud Computing

Cloud computing refers to as-needed, subscription use of off-site computer services, typically utilizing an internet connected network. Cloud computing allows organizations to rapidly adapt to and accommodate their changing computational needs. Cloud computing mitigates the often-substantial transitional financial and time lag costs associated with start-up or rapidly expanding computer needs. As the owners of the cloud computer services manage and maintain their product, subscribers avoid administrative and custodian cost burdens. Further, in the event of abrupt computational deceleration or change in operational goals, cloud computing eliminates organizational depreciation costs associated with dormant or obsolete equipment and software. Even stably established and well-resourced HPCCs may utilize cloud computing services to buffer acute fluxes in computer needs. Cloud computing currently plays a pivotal role in supporting the healthcare industry, including provision of the off-site storage of patient electronic medical records, the warehousing of large genomic data sets, the enablement of robust telehealth capabilities and the hosting of patient access portals [63]. Cloud computing utilizes the AI Silecosystem to automate complex healthcare data management protocols and enhance workflows associated with the processing and analysis of patient data [64]. Cloud AI platforms make more immediately available to oncologists and their patients the tremendous power of AI protocols [65]. AI-augmented cloud computing helps to advance tumor board operations, cancer therapeutics, patient management, diagnostics and oncology services [66].

##### Neuromorphic Computing

Neuromorphic computing adapts the physical architecture and functionality of the human central nervous system to enhance computer design and operation [67,68,69,70]. The artificial neuron constitutes the fundamental functional unit of neuromorphic computing. The construction and implementation of the artificial neuron and neuromorphic computers rely on interdisciplinary collaboration among neurobiologists, electrical engineers, computer scientists and computational specialists. Neuromorphic computing provided the basis for the invention and utilization of neuromorphic sensors such as artificial retinas and cochleae. Neuromorphic computing research inspired specialized subdisciplines, for example, neuromemrestive initiatives that utilize electromagnetic memristors to create CNS-computer interfaces [71]. Neuromorphic computing plays an ever-increasingly important role in healthcare applications such as patient safety monitoring [72], neuro-rehabilitation [73] and interactive health care robotics [74]. Recently, computer researchers have incorporated neuromorphic computing approaches into AI platforms to boost their effectiveness and efficiency [75,76,77]. Cancer scientists and oncologists have implemented AI-based neuromorphic computing to enrich their research [78,79,80] and improve clinical patient care [78,81].

##### Analog Neural Networks

As with neuromorphic computing, analog neural networks seek to mimic, more closely, the biochemical and neurophysiological functioning of the biological nervous system. Because biologic neuronal inputs comprise parallel converged signals originating from a multitude of neighboring neurons, the inputs do not occur within discrete time episodes, nor do the strength of signals have categorical quantitative values. Therefore, a nervous system model with analog continuous, rather than digital, input values more closely approximates actual nervous system functioning. Analog neural networks require less energy and less computational time compared with digital networks [82,83,84,85]. Analog neural networks now play central roles in the operation of numerous healthcare and medical software applications, e.g., those related to medical imaging [86], mimicking of the olfactory function [87] and modeling of mastoid bone pathologic events [88]. Investigators observe that analog neural networks may be used to support AI-based platforms such as vector machine learning [89], advanced edge computing [90] and natural language processing [91]. Cancer computational specialists have adapted analog neural networks to strengthen AI-informed oncology research, including the development of efficient cancer classification workflows [92,93], cancer histological analytic approaches [94] and oncology drug design pathways [95].

##### Monolithic-3D AI Systems

Electrical engineers originally designed the integrated circuit (IC) as a two-dimensional, flat semiconductor device containing a vast array of electronic elements such as transistors, capacitors and resistors. The IC has the capability to perform a wide range of data processing and computational operations. Relative to a collection of discrete circuit elements, ICs carry out operations more rapidly and use less energy. Recent advancements in IC design have led to the development of a three-dimensional (3D) IC configuration in which engineers vertically layer two-dimensional IC units [96]. This innovative design allowed construction of monolithic 3D ICs that contain within a single chip the necessary electronic components to carry out increasingly complex, advanced computational tasks [97]. Monolithic 3D ICs demonstrate improved efficiency of operation and allow for construction of ever more compact electronic instrumentation. The introduction of monolithic 3D ICs rapidly accelerated practical implementation of often very complicated AI machine learning and deep neural network algorithms in IoT devices such as personal, wearable medical devices and point-of-service health equipment [98].

##### The Graphics Processing Unit

The central processing unit (CPU) provides global program execution instructions for the computer; typically, the CPU performs its operational tasks in a serial fashion, one following another. CPUs normally contain a modest number of individual processing units (most often fewer than one hundred). Electrical engineers designed the CPU to complete dedicated large-scale computer operational tasks. In comparison, the graphics processing unit (GPU) has more limited operation execution responsibilities related to specific tasks [99]. The GPU can execute functions in a parallel fashion, handling multiple tasks simultaneously; facilitating parallel execution, the GPU may contain thousands of processing units. Although originally designed to perform video and graphics functions, computer scientists realized that vis-à-vis the CPU, the GPU performs AI-related tasks (e.g., machine learning and neural network operations) more proficiently. Oncologists have utilized GPU-based devices to augment their ability to implement radiation therapy [100] and interpret neuro-oncology MRI images [101]

##### Analog, Non-Volatile Memory Devices

Analog memory devices can store continuous data values. Volatile memory requires a continuous power source to retain data; non-volatile memory devices retain and stably store data after power discontinuation. The profound interest in implementing AI-based approaches, such as neuromorphic computing, that require durable and continuously valued data sets, has intensified the need for analog, non-volatile memory devices. Recently, engineers have innovated memory storage with the introduction of analog, nonvolatile ferroelectric field-effect [102,103], resistive random access memory [104,105,106], magnetic random access memory [107,108] and phase change memory technologies [109,110,111]. Analog, non-volatile memory has been instrumental in the continuing maturation of AI-based neural networks [84,112,113], image analytic platforms [114] and bio-sensor devices [115,116].

#### 3.3.2. Advances in Data

Data fuels the engine of the AI Silecosystem vehicle [117]; historically, several data-related innovations contributed to thawing of the AI Winters. Increasing the size of a data set characteristically elevates performance of an AI algorithm [118,119]. The advent of systematized large-scale data acquisition, concomitant with convergent informational and technical advances such as data compression [120], solid state memory [121] and random access memory [122], contributed to improved AI algorithmic functionality and abetted the awakenings of the AI Silecosystem from its early hibernations. In the following section, we examine additional data innovations that have driven forward the evolution and growth of the AI Silecosystem.

##### Synthetic Data

Synthetic data refer to information originating from an intentionally engineered process, in contrast to authentic data generated spontaneously from actual, real-world events. The desire for optimized AI algorithmic operability and larger data sets drove the development of synthetic data fabrication protocols.

Synthetic data production typically requires application of stringent statistical analytic procedures, precise data sampling approaches and rigorous testing methods to ensure accuracy and validity [123,124]. Synthetic data offer several key advantages over real-world data. For very large data sets, synthetic data avoid the often-tremendous financial costs associated with real-world data collection. Moreover, synthetic data, as they do not originate from actual patients, do not pose privacy risks and, additionally, eliminate the potential financial liability associated with a data breech. In addition, because of anonymity, synthetic data collections may allow their unrestricted use as open-source data repositories. The collection of real-world data may expose investigators to physical hazard. Data arising from natural disaster areas, associated with dangerous chemical or biologic agents, or originating from an unsafe physical environment (e.g., an active military combat zone or crime-challenged neighborhood) may all threaten the safety of data collection personnel. The surrogate production of synthetic data obviates such threats.

Within the AI Silecosystem, synthetic data have acquired increased prominence as recognition of their utility has grown. Synthetic data have driven forward innovations within the healthcare space. Synthetic data undergird many current initiatives in medical education [125,126], clinical training [127,128], epidemiology research [129,130] and disease prevention [131,132]. Cancer researchers now use synthetic data resources to bolster their work including precision medicine [133] and palliative care [134].

##### Facilitating Culturally Representative AI Data Sets

Experts identify cultural inequity and lack of diversity as ongoing and significant challenges in our society specifically impacting healthcare and medical outcomes [135,136,137]. As AI gains increasing currency as a tool to direct healthcare decision-making, and recognizing that patient data set composition influences AI algorithmic outcomes, consideration of the racial and ethnic composition of patient data sets has become important in order to ensure equity of healthcare outcomes, specifically within the sphere of cancer care [138]. Nevertheless, despite legal requirements for representative inclusion of racial and ethnic minorities in health research, disparities persist; data sets used in AI-based algorithms continue to employ non-representative patient populations, undermining the validity of algorithmic decision-making [139,140]. Novel initiatives aim to improve and maintain broad population representation within health care data sets and across AI platforms. These initiatives include the implementation of intentionally diverse data sets [141], the enactment of more effective legislative guidelines to promote equity and diversity [142] and initiation of proactive community programs to promote health research participation [143].

##### Optimizing Data Deposition and Engineering

In order to optimize functioning of the Silecosystem and performance of downstream applications, computer engineers and scientists require tractable access to high-quality, large-volume data [144,145]. For example, machine learning algorithms for drug discovery [146], diagnostic prediction [147] and oncology medical imaging [148] demonstrate significant improvement with enhancement of data quantity and quality. The construction of national federated data repositories seeks to establish direct, streamlined public access to large data warehouses [149,150,151,152,153]. Data engineering aims to modify and format data to facilitate AI model building and the completion of analytic tasks [154,155]. Recent data engineering efforts have sought to automate data quality improvement protocols such as eliminating bias in and assessing the integrity of large data sets [156,157,158].

Together, the careful generation of synthetic data, increased attention to equitable data representation and the facilitation of high-quality data access have promoted the saliency and amplified the currency of the AI Silecosystem. In the section that follows, we chronicle the role of software algorithms in mitigating past AI winters and their continuing role to solidify collective adoption of the AI Silecosystem.

#### 3.3.3. Advances in Software Algorithms: Piloting the AI Ecosystem

If hardware functions as engine, and data serve as fuel, then the software algorithm operates as pilot to direct the AI Silecosystem. As a pilot, the software algorithm directs the operational flow, direction and output of the AI Silecosystem. The AI computer scientist may choose among a variety of software algorithms; most frequently, the scientist utilizes machine learning or neural network algorithms [159,160].

Machine learning algorithms employ either supervised or unsupervised protocols [161]. With supervised protocols, input data have assigned labels that link with an output result; using this label, the algorithm then “learns” the rule that governs the relationship between the input and output data. With unsupervised protocols, the data lacks labels, and the algorithm must devise its own associative rules to understand patterns in the data. Among a range of practical applications, supervised machine learning has been used to predict customer behavior [162,163], differentiate cells of different histologies [164,165] and recognize faces [166,167]. With unsupervised machine learning, the algorithm seeks to cluster entities based upon some discoverable property of the entities, for example, grouping anonymous individuals within a large crowd based upon biometric or acquired physical variables [168,169].

Neural network algorithms, subsets of machine learning, generally supervised, work by mimicking the workings of the nervous system; within a neural network, an artificial neuron receives multiple inputs from neighboring neurons and then generates a resultant output based upon combined input [170]. In turn, the neuron transmits its output signal to other neighboring neurons, culminating, ultimately, in a final, consolidated output value from the system. The neural network algorithm “learns” the necessary rules that govern the correct association between input and output values. For example, computer scientists have adapted neural networking to interpret handwriting; this task entails making the correct association between a handwritten word and the ground truth, intended word [171,172,173].

Building upon the revolutionary impact of machine learning, other software inventions and algorithmic discoveries helped to rejuvenate AI and continue to transform the Silecosystem. A brief synopsis of major innovations follows.

##### Generative AI

Generative AI, an evolutionary offshoot of machine learning, uses rules derived from established instances of creative content to generate novel content such as original, advanced-level written documents [174], music compositions [175] and video game platforms [176], among others. Recently available generative AI applications, Microsoft’s ChatGPT [177] and Google’s Bard [178], have piqued the public’s attention as both tools demonstrate the ability to very quickly generate works that approach the imaginative and technical abilities of human creators [179,180]. ChatGPT and Bard have authored working computer code [181,182,183], achieved passing scores on professional qualifying and academic exams [184,185] and written jokes [186]. In the health care field, generative AI enables chatbot services [187], carries out natural language processing of medical records [188] and completes medical education tasks [189]. These generative AI applications currently play important roles in cancer drug discovery [190], review of cancer patient medical records [191] and digital pathology [192].

##### Virtual and Augmented Reality

Virtual reality relies upon AI-empowered three-dimensional viewing devices together with positional tracking to construct and allow participation in a simulated, pseudo-physical existence [193]. Augmented reality combines input originating from physical reality with information generated by a computer device to enrich the conscious experience [194,195]. Providers have utilized both virtual and augmented realities in health care, for example, to improve medical practice and basic science research, advance educational curricula [196,197,198,199,200], refine surgical skills [201,202], guarantee the safety and effectiveness of medical procedures [203,204] and alleviate cancer pain and suffering [205,206,207]. Future virtual and augmented reality efforts aim to optimize routine, everyday tasks as well as medical professional-related procedures [208,209,210].

##### Explainable Machine Learning

Machine learning algorithms achieve their solutions through progression of relationally dependent steps. The underlying logic governing these relations, however, may be abstruse and not readily decipherable by a computer scientist [211]. Disambiguating the machine learning logic yields significant benefits. For just as explaining the mechanism of a biologic process or chemical reaction may reveal secondary insights and lead to additional discovery, so also may explaining the logic of a machine learning solution lead to derivative AI computational breakthroughs [212]. Furthermore, end users of transparent, explainable machine learning algorithms have increased confidence in the predictions of and conclusion made by the algorithm [213,214]. AI computer scientists use a variety of explanatory methods to reveal and illuminate the underlying governing logic of a machine learning behavior [215,216,217,218]. For example, gradient methods quantify the effect that a change in a machine input parameter has on the algorithm output at each step of the algorithm [219,220]. Deconvolution protocols provide logical information about the logical relationship between a specific output feature and input variable [221,222]. Local interpretable, model-agnostic explanations work by randomly inactivating model inputs and then observing and collectively analyzing output results [223,224,225]. These and other explainable methods promise to enhance the intuitive utility of and confidence in machine learning as well as other AI-based methods. For example, oncologists have employed explainable machine learning to boost their ability to perform breast cancer morphological and molecular breast cancer profiling [226] as well as estimate cancer hospital length of stay [227].

##### Generative Adversarial Networks

Generative adversarial networks (GANs) represent a category of generative machine learning algorithms in which two neural networks, a generator and discriminator, “compete” to achieve a maximized generative outcome, for example, production of an artificial image indistinguishable from an actual image [228,229]. Ground truth data sets train the generator to produce artificial data and also train the discriminator to distinguish between actual and artificial data [230,231]. The GAN algorithm achieves its generative objective when the generator produces artificial data, a majority of which the discriminator fails to distinguish from authentic data [230]. GANs have applications across a variety of disciplines including natural language processing [232,233,234], cybersecurity [235,236], manufacturing [237,238,239] and military defense [240,241]. Prominently, science and medicine have adapted GANs to design and analyze biological networks [242], perform medical imaging [243,244], inform precision oncology [245] and prescribe radiation medicine protocols [246,247,248].

##### Neuro-Vector-Symbolic Architecture

Illustrative of the rapid transformation of the AI Silecosystem, computer scientists recently introduced a novel AI computer operational structure, neuro-vector-symbolic architecture (NSVA) [249]. NSVA combines two existing, highly impactful AI strategies, deep neural networks (DNNs) and vector symbolic architectures (VSAs). DNNs excel at discerning objects in images, but lack the ability to differentiate among similarly shaped objects with differentiating secondary characteristics [250,251]. VSAs have the capacity to distinguish among entities having a multitude of secondary characteristics; however, they faulter with image perception [252,253]. Thus, neither DNNs nor VSAs can independently solve image-based abstract reasoning problems adequately. NSVAs incorporate the strengths of both SVAs and DNNs without their inherent weaknesses to create an innovative AI architecture capable of solving complex, perceptual problems [254]. Applied architectural synergism, such as the NSVA, provides a model for evolving the AI Silecosystem to accommodate the burgeoning computational complexity brought about by the accelerated societal adoption and use of AI. Cancer specialists have adapted these novel architectures to aid image analysis [255] and tumor classification [256].

##### The Democratization of Resources/Open-Source AI Software

Open-source software refers to computer software universally available to individuals for unrestricted use, modification and distribution [257]. Open-source software, beyond facile, economic availability, accelerates computer discovery, engenders trust in the software and organically self-improves due to iterative public editing and optimization [258]. The AI community has access to a broad menu of open-source software applications. Two frequently used AI open-source programs, TensorFlow [259] and PyTorch [260], provide platforms for the development of machine learning programs. Computer scientists frequently utilize TensorFlow to develop and train deep neural networks [261,262]. PyTorch has a variety of uses including the construction of natural language processing applications [263,264] and image processing [265,266]. Open-source AI software promotes the free exchange of ideas among users, sustains the democratization and pace of AI Silecosystem maturation, and serves as a catalyst for continuing research, invention and insight. Currently, AI computer scientists employ open-source software solutions to facilitate brain cancer research [267], perform cancer digital pathology [268] and analyze cancer genomic data [269].

In Table 1 below, we provide a summary of the significant historical and ongoing hardware, data and software innovations with regard to their impact on seven key metrics of the AI Silecosystem: AI algorithmic speed, efficiency, utility, agility, accuracy, security and accessibility.

## 4. Tribulations of the AI Silecosystem: Impending AI Winter or Early Twilight of a Paradigm in Demise?

Interest in, adoption of and innovation associated with the AI Silecosystem have surged in no small measure due to the recent advances in the field of generative AI. With this surge, however, has come an amplification of concerns over the real and emerging risks and dangers of the AI Silecosystem [270]. Some experts see a more powerful AI Silecosystem as an existential threat to humanity [271]; the Center for AI safety recently advised that “mitigating the risk of extinction from AI should be a global priority alongside other societal-scale risks such as pandemics and nuclear war” [272]. Consequently, some societal leaders and countries have sought to pause or curtail continued AI development and/or use [273,274].

Regarding the use of AI within the healthcare and oncology sphere, leaders have voiced three broad concerns: loss of autonomy, malpractice and loss of compassion.

Scholars envision, on the horizon, ostensibly in the very near future, an AI singularity event wherein the intellectual capabilities of AI surpass that of humans, potentially with AI demonstrating unpredictable and uncontrollable behavior [275,276]. In this scenario, humans may unintentionally cede autonomy over their healthcare decision-making to an AI algorithm based upon actual superior medical insight [277,278,279], misperceived medical authority [280] or psychological manipulation [281].

Computer scientists and AI end users have expressed concerns over factual errors generated by AI algorithms [183,282,283,284]. AI-informed healthcare may pose real physical danger for the patient as AI algorithms may be prone to misdiagnosis [285] and incomplete or inaccurate treatment recommendations [286,287,288]. Healthcare specialists now recommend careful assessment of AI algorithms used for medical decision-making and expert review of AI-generated recommendations to avoid medical mistreatment [289,290].

Many patients do not trust AI [291,292,293]. Patients feel slighted by AI algorithms as the algorithms may, seemingly without apparent logic, deny patients health care coverage and needed services [294,295]. Patients perceive AI decisions as obdurate, unnuanced and arbitrary [296,297]. AI lacks compassion. The AI Silecosystem may be intelligent, but to many it is not wise.

These challenges, if not timely addressed, may precipitate the next AI intermission. Alternately, and potentially of greater consequence, the recent ascendancy of generative AI may presage an incipient twilight of the paradigm of “computer as thinking machine” along with the dawning of a succeeding, replacement paradigm, “computer as rational, sentient being”.

In Part I, we reviewed the primary hardware, data and software components of AI that enable its operation and advancement, encapsulated in the idea of the AI Silecosystem. As well, we chronicled the historical phases of progress and recession of the AI Silecosystem, conceptualized as the Kuhnian paradigm. In Part II that follows, we provide an example of practical utilization of the AI Silecosystem and illustrate its value to advance community oncology practice at the COH Comprehensive Cancer Center. We begin with a short discussion of the academic origins of the AI Silecosystem, and then proceed to detail its application at COH to advance community oncology practice.

## 5. The Academic Origins and Catalysis of the AI Silecosystem

The AI Silecosystem can trace its origins back to a number of key societal institutions that include commercial enterprises [298,299,300,301,302,303,304], the military [304,305,306,307] and, arguably, most prominently, academic centers [308,309,310]. Given their focus on research and education as well as their often substantial financial resources, academic centers became the natural home, incubator and accelerator of the AI Silecosystem. Because of their interdisciplinary and collaborative natures, academic departments often cross-pollinate ideas among departments and anticipate, react to and advance emerging paradigms such as the AI Silecosystem. Examples of notable AI advances originating from academic centers include invention of the Perceptron at the Cornell Aeronautical Laboratory in 1943 [311], conceptualization of the idea of AI at the 1956 Dartmouth Summer Research Project on Artificial Intelligence [312], construction of the first life-like robot at Waseda University in 1970 [313], demonstration of the first autonomous driving vehicle, the Stanford Cart, in 1979 [314] and creation of ImageNet, an annotated image repository, at Princeton University [315].

The emergence of the AI Silecosystem from academic centers accelerated adoption by academic healthcare and further advanced AI discoveries within the healthcare field. AI has established a widespread presence within medicine [316,317]. For instance, radiologists have harnessed AI to assist with interpretation of medical images [16,318,319], cardiologists use AI to diagnose and monitor patients with heart disease [320,321,322], gastroenterologists leverage AI to enhance the effectiveness of their interventions [323,324,325] and pulmonologists apply AI algorithms to optimize their diagnoses [326,327,328]. The AI Silecosystem has demonstrated tremendous value in oncology. Academic AI-based protocols have impacted oncologic approaches to the early diagnosis of cancer [329,330], targeted precision therapeutic recommendations [331] and palliative interventions [332,333]. After early applications in academic oncology, subsequent initiatives aimed to extend the AI Silecosystem paradigm to community oncology practice. Next, we chronicle these various initiatives.

## 6. Harnessing of the Academic Oncology AI Silecosystem to Advance Community Oncology Practice: The City of Hope Experience

**Figure 3:**
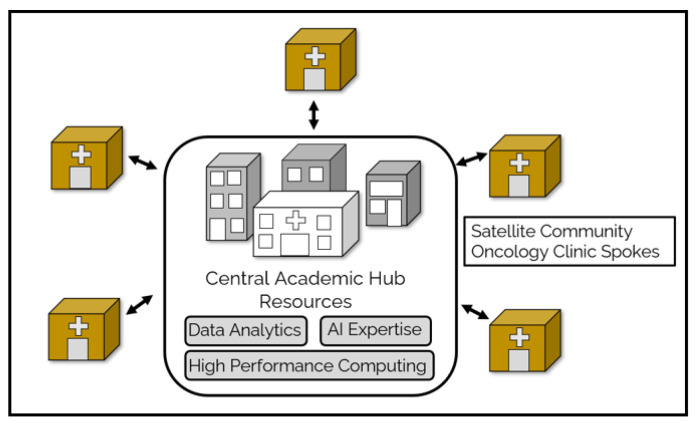
**Satellite COH community oncology clinics may access the institutional AI Silecosystem through hub-and-spoke service operations.** Community oncology practices may utilize data analytic, AI expert and HPCC resources via centralized network services provided to the COH community.

Although the AI Silecosystem has firm footing within academic oncology, its place within community oncology practice continues to mature. The City of Hope Cancer Center (COH) comprises a central, academic campus together with over 30 community satellite oncology practices. The central academic campus hosts COH’s AI Silecosystem. In the following section, we describe the hardware, data, and software algorithm resources of the COH Silecosystem, the availability of these resources to the community oncology practices and the efforts to advance AI-empowered oncology care within the COH oncology enterprise (Figure 3).

### 6.1. Hardware Resources: High-Performance Computer Cluster

To support AI computations, COH maintains a high-performance computer center (HPCC) comprising 7300 CPU cores, 80 TB of memory and 176 GPUs. All COH physicians, faculty, staff and students, including community oncology members, have privileges to access the HPCC remotely through desktop terminal applications. Round-the-clock IT experts provide technical support to assist with access to and utilization of the HPCC.

### 6.2. Data Resources

The COH Data Center manages and ensures reliable availability of several petabytes of deidentified clinical and genomic data for AI-related projects. To facilitate AI research and clinical projects, the Data Center relies on an institution-wide data repository, POSEIDON (Precision Oncology Software Environment Interoperable Data Ontologies Network), to house patient clinical and genomic data [334]. AI-assisted natural language processing organizes POSEIDON data according to a Common Data Model to optimize and accelerate downstream data input into AI operational workflows. To date, POSEIDON has assembled nearly one quarter million unique real world patient data sets. COH information and health care scientists have instituted and optimized operational protocols to structure efficiently patient-generated data for AI-based applications [335].

### 6.3. Software Resources

COH maintains a suite of bioinformatics and AI application modules on the HPCC. Clients may utilize HPCC resources and pursue AI investigations independently or collaboratively with COH expert consultants. COH established its Department of Applied Artificial Intelligence and Data Science (AAI/DS) to educate the COH community, facilitate institutional AI-based research and to provide clinical decision support to aid with AI modeling. AAI/DS hosts two forums each month. One forum, a journal club, reviews published manuscripts covering current areas of AI research including image analysis, machine learning and natural language processing. The second forum focuses on machine-learning-related institutional research initiatives, software applications and computational tools.

AAI/DS efforts have resulted in the creation of multiple machine-learning-based models to predict real world clinical events. Following bone marrow transplantation (BMT), the development of severe sepsis has an associated mortality rate exceeding 50%. One AAI/DS project utilized an ensemble approach combining multiple random forest binary classifications models to develop a tool to estimate the risk of patients developing life-threatening sepsis after BMT [336]. COH clinicians have employed this model to improve clinical care, avert sepsis-associated organ damage and ameliorate mortality events after BMT.

Serious complications such as cardiac events, pneumonia, hemorrhage and death many times follow cytoreductive cancer surgeries. Another AAI/DS initiative employed an explainable machine learning strategy to develop a model that predicts complications following cytoreductive surgery [337]. Surgeons at COH currently employ this model to identify patients at risk for post-operative complications and to implement preventive measures to mitigate these risks. For oncologists, time estimation until end of life in terminally ill patients poses a challenge; frequently, oncologists overestimate time until end of life. Such misestimation may negatively impact patient and family emotional and financial planning as well as confound medical management. Working with COH palliative care specialists, AAI/DS used a gradient-boosted trees binary classifier to create a model estimating time to end of life [338]. This model reliably outperformed oncologists for predicting 90-day mortality in terminally ill patients.

Alongside AAI/DS, associate COH departments and institutions further underpin the AI Silecosystem. The COH Center for Informatics, comprising the Divisions of Biostatistics, Clinical Research Information Support, Research Informatics and Mathematical Oncology, provides key computational support to the COH AI Silecosystem. The Center assists with the statistical design of research projects, restructures health and research data to be compatible with computer processing and aids with the visualization and analysis of data. AI projects supported by the Center for Informatics include the use of machine learning approaches to optimize, organize and structure electronic health care records for downstream artificial-intelligence-related projects [339], development of a machine learning platform to visualize and extract computationally employable information from biomedical and clinical data records [340] and utilizing machine learning approaches to advance the study and clinical implementation of immune-oncology [341].

The Translational Genomics Research Institute (TGen), a COH-affiliated center, leverages translational genomics to innovate diagnostic methods, molecular prognostic tools and targeted therapies for cancer through independent and collaborative projects [342]. Implementation of AI and machine learning algorithms have accelerated TGen-driven insights, fortifying the COH AI Silecosystem. One recent TGen-initiated scientific endeavor applied machine learning to develop a novel early cancer detection method, targeted digital sequencing (TARDIS) [343].

The cumulative energies of the AAI/DS, Center for Informatics, TGen, as well as the efforts of independent COH investigators have helped create a rich resource of AI expertise and maintain a robust portfolio of AI research. Examples of other initiatives at COHthat illustrate the depth and breadth of the AI Silecosystem include the use of AI autosegmentation for patients pending bone marrow transplant irradiation [344,345,346], AI-assisted oncologic drug design [347], expert critical review of clinical AI models [348], AI-based platforms for the evaluation and treatment of lung [349] and breast cancers [350], machine learning enabled pre-surgery physical status scoring [351] and AI-assisted irradiation dose estimation [352].

### 6.4. COH AI Silecosystem Engagement with the Community Oncology Network

Community Oncology patients and physicians at COH interface with and gain advantage from the AI Silecosystem on multiple levels. Every day, COH patients benefit directly from AI-informed institutional clinical care protocols such as the AI-informed diagnostic radiology, radiation oncology, medical oncology and palliative care initiatives described above. Moreover, community oncology patients may qualify for AI-based national clinical trials sponsored by COH. One such trial, currently available at COH, uses machine learning to inform the treatment of high-risk prostate cancer (NCT04513717) [353]. Community oncology patients also collaterally benefit from inclusion of their health care and genomic data in the electronic health record as their data help shape and make more accurate the AI models from which their AI-informed healthcare derives [354].

The COH AI Silecosystem likewise aids community oncologists. The AI Silecosystem provides access to expert AI specialists capable of providing to the community oncologist insights into the clinical serviceability and utilization of AI-based healthcare applications. Additionally, COH community oncologists may avail themselves of the many educational opportunities such as AI-related journal clubs, seminars and lectures. Further, COH community oncologists may employ the AI-Silecosystem data repository and institutional AI-associated hardware and clinical platforms for their own patient care [355]. Moreover, the COH AI Silecosystem helps expand AI-based clinical trial and research opportunities for community oncology providers.

## 7. Conclusions

The AI Silecosystem operates, innovates and advances as a synthesis of its component hardware, data and software elements. The AI Silecosystem has transformed in accordance with a Kuhnian paradigmatic progression with periods of rapid advancements punctuated by episodes of retreat. Recent signals of possible impending AI recession or even demise notwithstanding, the AI Silecosystem currently enjoys increasing societal currency and practical adoption. The academic oncology healthcare enterprise has significantly leveraged the AI Silecosystem to rapidly advantage cancer care, in particular the clinical management of the community oncology patient. The COH academic-community oncology team alliance demonstrates the practical feasibility and the tangible dividend of such leverage. In the near term, we may reasonably anticipate continued enthusiasm for the AI Silecosystem and its further utilization within community oncology practice.

## Figures and Tables

**Table 1:** Significant Past and Ongoing Advances in Hardware, Data and Software Driving Evolution of the AI Ecosystem and Their Value Impact.

				AI Silecosystem Metric						
Component		Innovation		AI Algorithmic Speed	Efficiency/Cost	Utility	Agility	Accuracy/Validity/Reliability	Security/Safety	Accessibility
Hardware		Quantum Computing								
		AI Internet of Things								
		Distributive Edge Computing								
		Cloud Computing								
		Neuromorphic Computing								
		Analog Neural Networks								
		Monolithic 3D AI Systems								
		Graphics Processing Unit								
		Analog Non-Volatile Memory								
Data		Synthetic Data								
		Culturally Representative Data Sets								
		Data Optimization								
Software		Generative AI								
		Virtual and Augmented Reality								
		Explainable Machine Learning								
		Generative Adversarial Networks								
		Neuro-Vector-Symbolic Architecture								
		Open-Source AI Software								
	Greater Impact			Lesser Impact						

## Data Availability

Not applicable.
